# Bacteriocins, Potent Antimicrobial Peptides and the Fight against Multi Drug Resistant Species: Resistance Is Futile?

**DOI:** 10.3390/antibiotics9010032

**Published:** 2020-01-16

**Authors:** Elaine Meade, Mark Anthony Slattery, Mary Garvey

**Affiliations:** 1 Department of Life Science, Sligo Institute of Technology, F91 YW50 Sligo, Ireland; Elaine.Meade@mail.itsligo.ie; 2 Mark Anthony Slattery MVB, Veterinary Practice, Manorhamilton, F91 DP62 Leitrim, Ireland; markslats@gmail.com

**Keywords:** bacteriocin, potency, resistant species, therapeutic, medical, efficacy, preservation

## Abstract

Despite highly specialized international interventions and policies in place today, the rapid emergence and dissemination of resistant bacterial species continue to occur globally, threatening the longevity of antibiotics in the medical sector. In particular, problematic nosocomial infections caused by multidrug resistant Gram-negative pathogens present as a major burden to both patients and healthcare systems, with annual mortality rates incrementally rising. Bacteriocins, peptidic toxins produced by bacteria, offer promising potential as substitutes or conjugates to current therapeutic compounds. These non-toxic peptides exhibit significant potency against certain bacteria (including multidrug-resistant species), while producer strains remain insusceptible to the bactericidal peptides. The selectivity and safety profile of bacteriocins have been highlighted as superior advantages over traditional antibiotics; however, many aspects regarding their efficacy are still unknown. Although active at low concentrations, bacteriocins typically have low in vivo stability, being susceptible to degradation by proteolytic enzymes. Another major drawback lies in the feasibility of large-scale production, with these key features collectively limiting their current clinical application. Though such limitations require extensive research, the concept of expanding bacteriocins from food preservation to human health opens many fascinating doors, including novel drug delivery systems and anticancer treatment applications.

## 1. Introduction

Antimicrobial drugs are undoubtedly one of the most important and useful therapeutic discovery’s in the history of medicine. Their implementation has allowed mankind to survive microbial disease, perform invasive surgical procedures, ensure animal health and protect the food chain. The discovery of the first antimicrobial agents Salvarsan, Prontosil and Penicillin was key in initiating the paradigms for future antimicrobial research. It was in the 1940s that the highly effective chloramphenicol was discovered and proved one of the most important antibiotics due to its potent bacteriostatic nature. Post the 1950s, the antibiotic era excelled with the discovery of new antibiotic classes at the forefront of research endeavors, the most prominent being the erythromycins followed by the glycopeptide vancomycin. Quinolones and Cephalosporins emerged in the 1960s, with the latter being divided into three generations relating to their spectrum of activity. Post the 1970s, however, no novel antibiotic agents have been discovered with the modification of existing drugs the best approach to combat emerging and re-emerging pathogenic species. The only exception being the relatively recently discovered teixobactin, an antibiotic exhibiting activity against methicillin-resistant *Staphylococcus aureus* (MRSA), *Streptococcus pneumoniae*, and mycobacteria [1]. During this period, antibiotic resistance was also emerging in bacterial species of medical importance. Methicillin was developed in 1959 as the first penicillinase-resistant β-lactam antibiotic for the treatment of beta lactamase producing staphylococcal species, with the emergence of MRSA less than a year later, still an ongoing health risk. Indeed, MRSA is recognised as one of the major risk pathogens associated with the development of antimicrobial resistance (AMR) [2]. 

Penicillin’s spectrum of activity and pharmacokinetics were improved with the induction of ampicillin in 1961 [3]. *Streptomyces clavuligerus* cultures provided the first bacterial β-lactamase inhibitors, which were used to derive clavulanic acid, subsequently combined with amoxicillin (amoxiclav) for the treatment of resistant species. As antibiotic agents are naturally produced by microbial species to gain an advantage in their ecological niche, antibiotic resistance so too has emerged from this microscopic war amongst the varied and diverse species of the microbial world. This explains the intrinsic resistance certain bacterial species possess for certain antibiotic agents with susceptibility to other classes evident. Acquired resistance, however, allows bacterial species to gain resistance mechanisms from neighboring species, subsequently increasing their arsenal of defense. As humans have increasingly implemented these therapeutic agents, the subsequent selective pressure has proliferated resistance amongst species, having dire medical consequences with multidrug resistant (MDR) bacterial infections increasingly common. ESKAPE organisms (*Enterococcus faecium*, *Staphylococcus aureus*, *Klebsiella pneumoniae*, *Acinetobacter baumannii*, *Pseudomonas aeruginosa*, and *Enterobacter* spp.), in particular, are emerging as extremely MDR [4]. Reports show an alarming trend where AMR infections result in 700,000 fatalities yearly, with 10 million deaths predicted by 2050 if this trend continues [5]. Effective prophylactic disinfection and sterilization of medical devices and medical fomites have successfully reduced the incidence of hospital acquired infections (HAIs). Additionally, the use of vaccination programmes aids in preventing the spread of contagious microbial disease also reducing the need for antibiotic disease treatment. Certainly, in order to combat the issue of AMR and MDR, novel approaches must be sought out to curb infectious disease, control pathogenic species, and ensure public health safety. The use of bacteria specific viruses termed bacteriophages, for example, shows real promise for the treatment of bacterial infections with phage therapy in clinical trials demonstrating efficacy [4]. Additionally, bacteriocins, bacterial ribosomal synthesized peptides with antibacterial activity, show promise as potent antimicrobial agents. This review aims to discuss the potential of these peptide toxins to act as antimicrobial therapeutics including sources, classification, and mode of action. 

## 2. Bacteriocins 

Bacteriocins are peptides produced by bacterial species in their ecological niches for self-preservation and competitive advantage. The majority of bacteriocins currently recognised are produced by Gram-positive bacteria with few known producers among Gram-negative bacterial species [6]. These small cationic molecules (30–60 amino acids), due to their amphiphilic helices, differ in their spectrum of activity, mode of action, molecular weight, and biochemical properties. A large portion of Gram-negative bacteriocins resemble eukaryotic antimicrobial peptides such as defensins [7]. These toxic peptides are classified according to their biosynthetic mechanisms as either ribosomally synthesized peptides demonstrating a quite narrow range of antimicrobial activity, and non-ribosomally synthesized peptides demonstrating a wider range of activity towards bacteria or fungi [8]. All major lineages of bacteria and archaea are believed to produce at least one bacteriocin. Typically, bacteriocins are very potent but have a limited spectrum of activity, only being effective on species that are phylogenetically related to the bacteriocin-producing bacteria itself [9]. At present, there are four classes (Class I to IV) of bacteriocins (Table 1), categorised based on the host producer, intrinsic function, molecular weight, physicochemical properties, and amino acid sequence. Bacteriocin synthesis genes are located chromosomally and on plasmids or transposons having minimum genetic machinery including structural associated immunity genes (immunity proteins) preventing self-toxicity. 

Bacteriocin synthesis is meticulously regulated, producing premature peptides with a leader sequence at the N-terminus. These toxic peptides are secreted in the bacterial logarithmic growth phase with increasing bacterial numbers promoting increasing peptide secretion [19]. Secretion is influenced by environmental factors including bacterial cell density, nutrient availability, the presence of acetic acid, and signalling peptides (competence stimulating peptide molecules).

### 2.1. Classes of Bacteriocins

Class I bacteriocins being less than 5 kDa are made up of small membrane-active, proteolysis- and heat-resistant peptides consisting of the amino acid lanthionine and methyllanthionine, which undergo posttranslational modifications and subsequent cleavage to generate the mature form. They include lantibiotics, sactipeptides, glycocins, and lasso peptides and are further grouped according to their structure and function into type A and type B [25]. Nisin A is the most common Class 1 bacteriocin and is ribosomally produced by strains of the lactic acid bacteria *Lactococcus lactis*. Antibacterial activity of Nisin A affects numerous Gram-positive genera such as *Staphylococci*, *Streptococci*, *Listeria*, *Bacilli*, *and Enterococci species* and are typically inactive against Gram-negative species. Nisin A and its variants are the main representatives of lantibiotics with other members of this class consisting of lacticin 481, carnocin U149, and lactosin S and the *Bacillus* peptides subtilin and subtilosin A. Staphylococcins are bacteriocins produced *Staphylococcus* species belonging to classes I, II, and III with lantibiotics the predominant class I and aureocin A70, aureocin A53 and epidermicin NI01 of the class II type [26]. 

Class II bacteriocins (<10 kDa) consist of four subtypes (pediocin-like, two-peptides, circular and nonpediocin-like linear) and are small non-lanthionine containing, unmodified, membrane active, temperature and pH resistant polypeptides. Anti-listeria, pediocin-like peptides such as pediocin PA-1 constitute subclass IIa with bacteriocins that require two or more peptides for activity constituting subclass IIb (lactacin F, Lactococcin M, Lactococcin G). Leaderless bacteriocins, without an N-terminal leader peptide (lactococcin B), are grouped in subclass IIc with unmodified non pediocin-like linear bacteriocins grouped in subclass IId Class IIc bacteriocins are commonly referred to as circular bacteriocins relating to the covalent linkage of the N- to C-termini giving a compact circular structure enabling their temperature and pH stability. Indeed, circular bacteriocins produced by Gram-positive bacteria of the phylum Firmicutes are located in the gastrointestinal tract (GIT) as part of the human microbiota where they have an immunomodulating role. The circular antimicrobial peptide subtilosin A, produced by *Bacillus subtilis*, was initially classed as a circular bacteriocin. Subtilosin A and the sporulation killing factor (SKF) of *Bacillus thuringiensis*, however, contain thioether linkages and are now recognised as structurally and genetically separate from circular bacteriocins [27]. *Enterococcus faecalis*, *Lactobacillus species*, *Clostridium beijerinckii*, *Carnobacterium maltaromaticum*, *Butyrivibrio fibrisolvens*, *Streptococcus uberis*, *Lactococcus species* and *Leuconostoc mesenteroides* are all known producers of circular bacteriocins [26]. 

Class III bacteriocins (helveticin J, helveticin V-1829, lysostaphin, lactacin A and B, acidophilus A) are large (>30 kDa) heat-labile proteins. Lysostaphin is a staphylococcin bacteriocin. Colicin and microcins are class III bacteriocins produced by the Gram-negative species, *Escherichia coli* [28] with pyocin and salmocins produced by *Pseudomonas aeruginosa* and *Salmonella* species, respectively, where class IV bacteriocins are complex proteins that require essential lipid or carbohydrate conjugation to enable activity [29]. Some reports, however, classify these protein-macromolecule complex’s as bacteriolysins (hydrolytic polypeptides), giving three bacteriocin classes [8]. Indeed, the bacteriocin classification groupings are continuously evolving as more information on their individual specifics is gathered describing their intricacy and diversity. 

### 2.2. Mode of Action

Antimicrobial peptides (AMPs) are produced by nearly all living organisms and exhibit their activity via different mechanisms of action including binding to genetic material, interaction with the cell wall, interaction with the cell membrane and interaction with intracellular organelles [30]. AMPs are believed to be essential in protecting plants from invasive fungal and bacterial species. Certain invertebrate AMPs and vertebrate possess activity against parasite and viral species [31]. Such potency, however, may lead to toxic effects on host cells as AMPs can also target eukaryotic cells. As most bacteriocins are cationic and smaller than 10 kDa (except class III bacteriocins), their small size, charge and alteration in hydrophobic and hydrophilic properties allows them to adhere to microbial cells and penetrate phospholipid membranes [32] with prokaryotic selectivity a clear advantage over AMPs. 

As such, the antibacterial activity of lantibiotics (e.g., nisin) results from the formation of pores in bacterial cell membranes. These elongated amphiphilic cationic peptides induce the formation of pores in negatively charged bacterial cell membranes resulting in an efflux of small metabolites from susceptible cells. Others such as nisin Z (a variant of nisin A) exerts activity by binding to a specific target (docking molecule), membrane-anchored cell wall precursor lipid II, which is also targeted by the glycopeptide vancomycin [33] having effect at nanomolar concentrations. Similarly, subtilin binds a target molecule, bactoprenyl pyrophosphate, to permeabilize the target cell membrane in a lipid II-dependent manner, while mannose phosphotransferase proteins IIC/D operates as the docking molecule for class IIa bacteriocins [32] with a zinc-dependent membrane-bound protease of the M50 family being the target of numerous related leaderless bacteriocins [27]. Subsequent membrane permeabilization induces cellular leakage of ions and metabolites, depolarization of the transmembrane potential and consequently reduced osmotic regulation, inhibition of respiration and finally membrane rupture and rapid cell lysis [30]. Additionally, membrane permeabilization allows for translocation of toxic peptides into the cytoplasm where cell organelle may be targeted influencing DNA/RNA integrity, protein synthesis, cell wall synthesis and enzyme activity. The outer membrane of Gram-negative species protects them from bacteriocins produced by Gram-positive species. Under stress conditions, however, such as acidic pH, temperature variations, presence of chelators, absence of metal ions and high saline environments, certain Gram-negative species including *E. coli* [34] and *Salmonella* become sensitive to Gram-positive bacteriocins [6]. 

Recent studies show that Helveticin M can disrupt the cell wall of Gram-positive bacteria and outer membrane of Gram-negative bacteria, having efficacy to both bacterial categories [28]. Disruption of the outer membrane of Gram-negative bacteria by the metal chelating agent ethylenediaminetetraacetic acid (EDTA) aids in the antimicrobial activity of the class II bacteriocins carnocyclin A [27], an effect also observed with nisin. The globular lantibiotics (e.g., mersacidin) of class I bacteriocins act via enzyme inhibition on essential enzymes of the target cell [9], whereas class III bacteriocins have antibacterial activity via the lysis of sensitive cells by catalysing cell-wall hydrolysis [33]. Gram-negative colicins (large peptides) and microcins (small peptides) possess several cytotoxic mechanisms, such as pore formation, degradation of peptidoglycan precursors, phosphatase activity, activity targeting 16S rRNA and specific tRNAs and DNAse activity [33]. Indeed, the antibacterial activity of AMPs is likely a result of multiple actions inducing a multi-hit mode of toxicity on the target organisms. Furthermore, some bacteriocins including nisin and enterocin (produced by *Enterococcus faecalis*) have also demonstrated sporicidal activity against the spore forming *Clostridium botulism* and the thermophilic spore forming *Bacillus cereus* and *Geobacillus stearothermophilus* [24]. While the antimicrobial activity of bacteriocins has been recognized for some time, the exact mode of action for many of these peptides has yet to be elucidated. 

### 2.3. Bacteriocin Resistance Mechanisms

As bacterial species display resistance to antibiotics, they may also demonstrate resistance to AMPs and bacteriocins which is chromosomally located or acquired. Resistance may develop by mimicking the natural defense immunity of the producer strains, enzymatic degradation of the bacteriocin, adapting the cell membrane and growth conditions [32]. Furthermore, as bacterial membrane surface charge and membrane variability are key factors in bacteriocin toxicity, alteration of these factors promotes bacterial resistance. Mimicking the natural defense immunity occurs when non-bacteriocin-producing strains possess genes homologous to the self-protecting immunity genes of bacteriocin producers. Enzymatic degradation results when assaulted bacterial species excrete enzymes that degrade bacteriocin peptides; nisinase is one such defense molecule produced by *Bacillus cereus* and *Paenibacillus polymyxa* [35] causing nisin degradation. Alterations in surface charge of the cell wall due to gene mutations disrupt bacteriocin binding, providing resistance to nisin and pediocin in *Listeria* species [32] depending on the phase of the growth cycle. Resistance to nisin has been observed in *Clostridium botulinum* with some strains also showing cross resistance to class II bacteriocins [24].

Similarly, to antibiotic resistance, bacteriocin resistance from gene mutations or horizontal gene transfer (HGT) via transformation, conjugation, or transduction has an effect by altering the cell wall, cell membrane, receptors, and essential systems. This raises concern if bacteriocins were to be used in conjunction with antibiotic therapeutics as cross resistance may occur, producing multi drug and bacteriocin resistant variants. Recent studies have indicated that bacteriocin resistance can be defeated using a multi-hit combination of bacteriocin types and bioengineering techniques providing increased functionalities. Indeed, bioengineered variations of nisin has provided variants demonstrating more potent activity against AMR species including MRSA, vancomycin resistant *Enterococci* (VRE) and *Clostridium difficile* while also proving efficacy against some Gram-negative species [36] strengthening their use in medical applications.

## 3. Medical Applications of Bacteriocin Therapeutics

At present, the global health crisis predominately relates to two main issues: (1) the increasing rates of morbidity and mortality due to cancer and (2) the spread of infectious disease particularly with AMR pathogens. Indeed, infectious disease in immunocompromised oncology patients significantly increases the duration and severity of morbidity. Furthermore, studies report chronic infection resulting from *S. aureus, K. pneumonia, A. buamannii*, and *P. aeruginosa* increase the risk of cancer development due to a reduced immune function in some persons [37]. Novel approaches are needed to prevent and treat these life-threatening conditions as traditional therapeutic options continuously prove ineffective. 

### 3.1. Infectious Disease

Lantibiotics exhibit potent activity against Gram-positive bacteria including clinically relevant AMR and MDR species including MRSA, VRE, vancomycin intermediate *S. aureus* (VISA), *Streptococcus pneumoniae, Listetria*, *Bacillus sp.* and *Clostridium difficile*. As such, several lantibiotic peptides displaying outstanding in vivo activity have been put forward clinically for the treatment of potentially fatal bacterial diseases [36]. Furthermore, the lantibiotic mutacin B-Ny266 displays activity against Gram-negative strains of Neisseria and Helicobacter with purified nisin demonstrating activity against *E. coli* [6]. Extended-spectrum β-lactamase (ESBLs) producing Gram-negative bacteria are of great concern due to their intrinsic resistance to most β-lactam antibiotics including penicillin’s and cephalosporins. Additionally, infections with shiga toxin-producing *E. coli* (STEC) and ESBL *Enterobacterales* are a global health concern [34] as infections with drug resistant nosocomial pathogens often have dire consequences. As Gram-positive bacteriocins are predominantly ineffective against Gram-negative species, perhaps the hope for targeting these pathogens lies in the peptide toxins produced by the ESBL *Enterobacterales* themselves. The Gram-positive *Enterococcus faecalis* is also a nosocomial pathogen of great concern, commonly associated with endocarditis, urinary tract, and systemic infections. This species and its variant VRE are known to possess resistance to many classes of antibiotics including aminoglycosides, daptomycin, quinolones, macrolides, rifampicin and β-lactams [1]. 

The use of bacteriocins incombination with antibiotics has shown efficacy at reducing the concentration of antibiotic needed (chloramphenicol amongst others) to inhibit bacterial cell growth of *E. faecalis* [38]. Nisin, for example, displays potent anti-biofilm activity against *E. faecalis* in combination with penicillin, ciprofloxacin, and chloramphenicol [39], a find also reported by Tong et al. [38]. Findings also demonstrate the anti-biofilm activity of nisin in combination with polymyxins against Gram negative *P. aeruginosa* [1]. A significant find as biofilm structures are extremely chemical and antibiotic resistant resulting in increased mortality and prolonged infection in patients. The formation of biofilms on medical devices including catheters is extremely problematic where they constitute the most frequent cause of nosocomial septicemia. Bacteriocins Pep 5 and epidermin produced by *Staphylococcus epidermidis* both demonstrated an inhibitory action against the adhesion of *Staphylococcus* species to the surfaces of silicon catheters [36]. Additionally, nisin has been reported to possess biofilm penetrating abilities potentially making it a useful tool in preventing or eradicating biofilm communities [40] on invasive medical devices. Preliminary investigative studies also suggest possible uses of nisin as antibacterial coatings on implantable medical devices [40]. In vivo animal studies have demonstrated the efficacy of lantibiotics for preventing and treating infectious disease. Nisin F proved effective against *S. aureus* pulmonary infections in a rat model, with mersacidin eradicating MRSA colonization in a mouse rhinitis model and lacticin demonstrating efficacy against systemic *S. aureus* in a mouse model [41]. While the treatment of cystic fibrous (CF) patients with inhaled pyocins to elevate *Pseudomonas* colonisation or vaginally delivered colicins to treat *E. coli* UTIs may offer plausible options to control these common infectious diseases. Reports indicate, however, that pyocin did not control *Pseudomonas* lung infections in patients with cystic fibrosis despite promising in vitro findings [42]. Specifically, the findings of Ghoul et al. show a lower bacteriocin diversity correlating with the ability of *P. aeruginosa* to persist in infections of the CF lung coupled with several immunity systems present in this species [43]. Nisin variants are currently being implemented as antibacterial sanitizers to control pathogenic *Staphylococcus* and *Streptococcus* species associated with mastitis in lactating cows [7]. Research using recombinant PCR techniques to integrate enterocin CRL35 and microcin V genes, producing the combined bacteriocin Ent35-MccV allowed for activity against clinically isolated enterohemorrhagic *E. coli* and *Listeria monocytogenes*, amongst other pathogens [22]. 

While the benefits of bacteriocins in treating infectious disease appear evident, it must also be noted that some Gram-positive bacteria can utilize bacteriocins as virulence factors increasing their pathogenicity. One such example includes the enterococcal lantibiotic cytolysin which possess cytotoxic activity against a wide range of cell types, including Gram-positive bacteria and human, bovine and horse red blood cells, retinal cells, polymorphonuclear leukocytes and human intestinal epithelial cells, and is associated with acute and terminal infectious disease in humans [32]. Similarly, pathogenic streptococcal strains produce various bacteriocin virulence factors including the hemolysins intermedilysin and streptolysin S, involved in invasive group A *Streptococcus* infection [44]. 

### 3.2. Anti-Cancer Activity

Malignancy remains one of the most difficult to treat and serious diseases globally, resulting from unscheduled and unregulated cell proliferation. Furthermore, the methods of cancer treatment in recent decades including surgery, radiotherapy and conventional chemotherapy have not greatly reduced the rate of mortality in patients [45] with significant side effects present. Currently there is a requirement for a paradigm shift in treatment options with novel approaches at the forefront of innovative needs. Recent studies have indicated the activity of bacteriocins against some tumor cell lines in vitro [22]. Colicin A and E1 produced by *E. coli* demonstrated growth inhibition of human fibroblast, bone, breast and colon cancer cells amongst other cell lines in vitro [46]. Colicin D and E3 was shown to induce a dose dependent inhibition of murine leukemia cells at 0.4, 0.8, 1.6 and 3.2 mg/mL in vitro [36]. Hertz et al. report the in vitro anticancer activity of microcin E492 on colorectal and breast cancer cells at 5, 10, and 20 μg/mL via apoptosis and necrosis at concentrations of 10 and 20 μg/mL [47]. In vivo mice studies demonstrated the ability of nisin in controlling head and neck squamous cell carcinoma and oral cancer [42]. Notably, additional purified bacteriocins, including pyocin, colicin, pediocin, and microcin, have also displayed inhibitory properties against neoplastic cell lines and in xenograft mouse models [48]. 

Bacteriocins with anticancer activity appear to be cationic, amphiphilic and membrane active with cytoxicity due to necrotic cell membrane lysis induced by increased presence of negatively charged molecules on the membrane surface [7]. Additionally, cancer cells have higher membrane fluidity compared to normal cells allowing for membrane destabilization [46]. Moreover, other bacteriocins are known to affect angiogenesis, subsequent cancer progression, disrupt the integrity of mitochondrial membrane and induce apoptosis and cell cycle arrest in cancer cells [49]. Cell death by apoptosis is more favorable as it does not induce an inflammatory response as observed with necrosis. Cancer cell membranes are mainly negatively charged as they contain anionic phosphatidylserine, O-glycosylated mucins, sialylated gangliosides, and heparin sulphates allowing for cancer selectivity as normal cells are neutral in charge [50]. Bacteriocins also produce an immune modulatory response against T and B cells involved in the control of cancerous pathways, stimulate cytokine secretion and modify the tumour microenvironment increasing the efficacy of cancer treatment [42]. 

### 3.3. Factors Affecting Medical Application

Bacteriocins offer many advantages for therapeutic application including their small size, biocompatibility, biodegradable and mostly non-immunogenic nature [45]. Issues arise, however, relating to bacteriocin stability, solubility, large-scale production and purification in sufficient quantities for general medical use. Solid or liquid phase synthesis and in vivo biotechnology recombinant methods are typically used for the production of medical peptides. Due to the complex nature of bacteriocin peptides and the need for posttranslational modification however, such synthesis methods are costly and unrealistic for large scale production [40]. Furthermore, an additional issue with chemical synthesis is the long and/or hyper-hydrophobic peptide design that can self-aggregate, obstructing the elongation steps [25]. 

Orally administered bacteriocins are susceptible to enzymatic and pH degradation in the GIT with pharmacokinetics such as intestinal absorption, bioavailability, distribution, half-life, renal clearance and elimination, all important factors requiring investigation. Bacteriocin peptides are expected to have a lower half-life than their antibiotic counterparts [25] due to their sensitivity to proteases in vivo. Studies conducted on chemically synthesized bacteriocin peptides integrating d-amino acids, however, show that these peptides are less susceptible to proteolytic cleavage in the GIT [50]. While Shea et al. produced a trypsin resistant variation of salivaricin P via alteration of trypsin recognition sites having slightly reduced activity against *Listeria* species [51]. Parenteral administration may offer some means of avoiding proteolytic degradation of bacteriocins [52] particularly in cases where systemic infections are present. Parenterally administered bacteriocins will be in contact with proteases involved in hemostasis and fibrinolysis in the blood stream [1] and this may reduce their activity. Studies assessing peptide modification of bacteriocins may provide structural information to improve this issue perhaps by removing proteases recognition sites.

In comparison to antibiotic drugs, bacteriocins are less labile at high temperatures and in extreme pH environments. Bacteriocin stability relates to their diverse structure and level of post-translational modifications (cyclization, disulfide bridges, and nonconventional amino acids) needed for activity [25]. Bioengineered variants of natural bacteriocins have displayed increased efficacy with studies describing bioengineered mutants of nisins A and Z having modulated pharmacokinetic properties [47]. In terms of biocompatibility, class II bacteriocins, nisin, and other lantipeptides, have proved to be non-cytotoxic to various eukaryotic cell lines at doses 100-fold higher than antibacterial concentration [25]. Further studies are necessary to establish a means of improving stability and potency of bacteriocins for medical applications (Table 2). Additionally, studies investigating the precise mechanisms of bacteriocin resistance will expedite their application in clinical settings individually or as combination antimicrobials [1].

## 4. Food Applications

Microbial foodborne disease remains an ongoing issue globally, proliferated by the increasing human population and demand for healthy sustainable food resources. Pathogenic and food spoilage organisms cause consumer morbidity/mortality, economic impacts and large volumes of food waste. Numerous pathogens exist in human food systems, both as planktonic and biofilm cells. Bacterial species such as the Gram-negative *Salmonella enterica*, *Escherichia coli*, *Campylobacter jejuni*, and the Gram-positive *Listeria monocytogenes* [51,53] and *Clostridium perfringens* are common foodborne pathogens with foodborne outbreaks increasing globally [5]. Protecting the food chain from microbial contamination at all stages of production is vital to ensure public health safety [54]. Furthermore, disease prevention and control in livestock animals is essential to eradicate food contamination. Indeed, the widespread use of antibiotics including tetracyclines, penicillin, macrolides, sulfonamides, aminoglycosides, and cephalosporins in food-producing animals has undoubtedly proliferated the issue of AMR and MDR in animal pathogens with zoonotic transmission a serious issue [55]. For example, in swine food production, isolated strains of enterotoxigenic *Escherichia coli* possessed resistance to tetracyclines, aminoglycosides, trimethoprim–sulphonamides, and ampicillin with *E. coli*, *Salmonella*, *Enterococcus and Clostridium* strains showing resistance to tetracycline, ampicillin and streptomycin frequently isolated from broiler chickens [41].

Bacteriocins have been successfully implemented as food preservatives with nisin “generally regarded as safe” (GRAS) by the Food and Drug Administration (FDA) where it is used to prevent to the germination of *C. botulinum* spores in cheese products [40] and growth of *L. monocytogenes* in refrigerated dairy [56]. Studies also described the activity of nisin against *S. aureus*, *B. cereus* and *B. subtilis* in processed cheese [57]. Indeed, biologically produced nisin has been successfully implemented as a food preservative for over five decades without displaying microbial resistances [39]. Pediocin shows the potential to inhibit the growth of *L. monocytogenes* in readymade food products. Carnocyclin A marketed as Micocin in the US and Canada has been developed to inhibit *Listeria monocytogenes* in ready-to-eat meat products [58]. The semipurified bacteriocins BacTN635 (*Lactobacillus plantarum* sp. TN635) and BacFL31 (*Enterococcus faecium* sp) have demonstrated activity against *L. monocytogenes* and *Salmonella typhimurium* in poultry and beef while also improving the organoleptic properties of the meat such as color and odor [59]. *Streptococcus suis* is a Gram-positive pathogen associated with severe infections and economic losses in food producing pigs. Importantly, *S. suis* is currently recognized as an emerging zoonotic pathogen and high risk for persons exposed to infected pigs or their by-products, often displaying resistance to macrolides, tetracyclines and colistin [60]. Nisin excreted by *L. lactis* (ATCC 11404) demonstrated antibacterial activity toward *S. suis* in vitro with purified MIC values of nisin ranging from 1.25 to 5 μg/mL [41]. Bacteriocins used in food products help meet consumer demand for high quality non chemically processed safe food [56]. Bacteriocins, however, may also be used in combination with other preservation methods (termed hurdle technology) to increase the shelf life and safety of the food items. For example, nisin used in conjunction with amoxicillin and ceftiofur showed increased activity against *S. suis* in vitro [41].

The control of *Campylobacter jejuni* in poultry remains an ongoing issue for public health safety with the incidence of *Campylobacter* infections increasing worldwide [59]. The addition of bacteriocins to poultry feed may aid in reducing the presence of this robust species in the GIT of bird’s pre-harvest. Studies have shown that feed incorporating bacteriocins enterocin E-760 and B602 (*P. polymvxa*) encapsulated in polyvinylpyrrolidone decreased the intestinal population of *C. jejuni* in poultry [41]. Pure bacteriocins and probiotics containing bacteriocin producer strains can alter the intestinal microbiota of animal species (including human) reducing the number of pathogens present. This may not always have a beneficial effect however, as dysbiosis, a negative imbalance of the GIT microtia is associated with numerous disease states both local and systemic. Furthermore, in food items following consumption, the activity of bacteriocins may be diminished by adherence to food particles, proteolytic enzyme activity in the GIT, pH variances and interaction with the human GIT microbiota. For use in food products, therefore, these peptides must possess some essential properties such having a broad spectrum of activity, be safe for consumers (GRAS), heat and pH stable and be soluble and stable in the food matrix. The use of bacteriocins in food packaging has also gained recognition as a potential means of controlling food spoilage where studies have shown that incorporating bacteriocins into packages has advantages over conventional methods such as controlled release of the peptide on to the product with limited interaction with the food item [57].

## 5. Conclusions

As the incidence of antibiotic resistant infections increases globally from disease associated with nosocomial and foodborne pathogens, there is an urgent need to develop novel antimicrobial agents. If possible, novel antimicrobial substances should possess different modes of action to currently used therapeutics in order to effectively combat resistance in pathogenic species. Novel substances which can be used alone or in combination with antibiotics reducing the dose required for activity are of the upmost importance. Bacteriocins show potential in this context as they exhibit multiply modes of action by forming pores in membranes, inhibiting cell wall biosynthesis and affecting cellular respiration. Additionally, some bacteriocins, notably nisin, have demonstrated sporicidal activity, activity against Gram-negative species and anti-biofilm activity, further demonstrating their potential clinical importance. Due to their ribosomally synthesised nature, bacteriocins are very amenable to bioengineering with bioengineered variants demonstrating increased potency to select bacterial strains while having low levels of toxicity to animal cells. Further studies are warranted to determine suitable delivery mechanisms as proteolytic digestion in the intestinal tract may affect bacteriocin activity. Novel drug delivery mechanisms may overcome this issue with parenteral delivery a plausible option. Bacteriocins also demonstrate anti-cancer activity in vitro with efficacy in the micromolar range and selectivity to cancerous cells. Changes in membrane charge and fluidity due to enhanced expression of negatively charged cell surface molecules appears to allow for selectivity. Additionally, cell death also appears related to apoptosis and depolarisation of the membrane in in vitro studies. Consequently, the in vivo investigation of the anticancer and antimicrobial efficacy of bacteriocin peptides to treat local and systemic disease appears warranted.

## Figures and Tables

**Table 1:** Classes of bacteriocins categorised based on the host producer, intrinsic function, molecular weight, physicochemical properties and amino acid sequence.

Bacteriocin	Host Producer	Intrinsic Function	Mol. Mass (kDa)	No. A. Acids	Physiochemical Properties
**Class I: Ripps—Ribosomally synthesized Post-translationally modified Peptides**					
**Heat stable, lanthionine and methyllanthionine containing peptides (<5 kDa)**					
**Lantibiotics**					
**Subtype A1: Leader peptides are cleaved by a dedicated serin proteinase**					
Microbisporicin	*Microbispora corallina*	Bind to a docking molecule, either inhibiting cell wall synthesis or forming pores in the cell membrane	2.2	24	Modified by LanB (dehydration) and LanC (ring formation). Exported by LanT and released from leader peptide by LanP [10] Elongated, linear, flexible, amphipathic molecules
Nisin A/Z	*Lactococcus lactis*		3.4	34	
Pep5	*Staphylococcus epidermidis*		3.3	34	
Subtilin	*Bacillus subtilis*		3.3	32	
**Subtype A2: Leader peptides are cleaved by a dedicated ABC ATP-binding cassette [ATP] transporter**					
	*Carnobacterium piscicola*	Bind to a docking molecule, inhibiting cell wall synthesis	4.6	35–37	Modified by LanM (bifunctional—dehydration and ring formation). Transported and processed by LanT [10] Globular, negatively charged or neutral molecules.
Lacticin 481	*Lactococcus lactis*		2.9	27	
Plantaricin C	*Lactobacillus plantarum LL441*		2.9	27	
**Subtype B**					
Actagardine	*Actinoplanes liguriae*	Bind to a docking molecule, inhibiting cell wall synthesis	1.9	19	
Mersacidin	*Bacillus sp. strain HIL Y-85,54728*		1.8	19	
Lacticin 3147 (LtnA1 and LtnA2)	*Lactococcus lactis subsp. lactis DPC3147*	Bind to lipid II, inhibiting cell wall synthesis or forming pores [11]	4.2	59	
**Sactipeptides**					
Subtilosin A	*Bacillus subtilis*	Not completely understood	3.4	32	Peptides with cysteine sulfur to α-carbon crosslinks which are catalyzed by radical S-adenosylmethionine (SAM) [12]
Thurincin H	*Bacillus thuringiensis* SF361		3.1	31	
Thuricin CD (Trn-R and Trn-β)	*Bacillus thuringiensis*		2.8	30	
**Glycocins**					
Glycocin F	*Lactobacillus plantarum*	Bacteriostatic—Little know	4.0	43	Glycosylated antimicrobial peptides [13]
Sublancin 168	*Bacillus subtilis*	Bactericidal—Affects protein and DNA synthesis	3.7	37	
**Lasso Peptides: An N-terminal macrolactam with the C-terminal tail threaded through the ring**					
**Subtype I**					
Siamycin-I	*Streptomyces* spp.	Inhibition of cell wall synthesis	2.1	21	two disulfide bridges linking the macrocyclic ring with the threaded tail
Aborycin	*Streptomyces* spp.		2.1	21	
**Subtype II**					
Capistruin	*Burkholderia thailandensis E264*	Inhibition of RNA synthesis [14]	2.0	19	Contain no disulfide bridge
Microcin J25	*Escherichia coli*		2.1	21	
Klebsidin	*Klebsiella pneumoniae*		2.0	19	
**Subtype III**					
BI-32169	*Streptomyces* spp.	Glucagon receptor antagonist [15]	2.0	19	one disulfide bridge that links the N-terminal ring and the C-terminal tail
**Subtype IV**					
LP2006	*Nocardiopsis alba*	Not completely understood	2.0	17	one disulfide bridge that links the C-terminal tail to itself [14]
**Class II: Unmodified peptides**					
**Heat-stable, non-lanthionine containing bacteriocins (<10 kDa)**					
**Subtype IIa: Pediocin-like peptides**					
Pediocin PA-1	*P. acidilactici PAC1.0*	Membrane active—Disrupt the proton motive force of the target cell by pore formation.	4.6	44	Linear peptides which contain a highly conserved hydrophilic and charged *N*-terminal region that has a disulphide bond linkage and a consensus sequence of YGNGVXC [16]
Leucocin A	*Leuconostoc geldium UAL 187*		3.9	37	
Enterocin NKR-5-3C	*Enterococcus faecium NKR-5-3*		4.5	43	
Microcin L	*Escherichia coli*	Disruption of cell membrane [17]	8.9	90	Plasmid-mediated, contain disulfide bonds but no further posttranslational modification [18]
Microcin N/24	*Escherichia coli*	Unknown	7.3	73	
**Subtype IIb: Two-peptides**					
Lactacin F	*Lactobacillus acidophilus*	Disrupt the proton motive force of the target cell by pore formation.	6.3	57	Mostly cationic peptides. Requires synergy of two different peptides to form an active poration complex [16]
Enterocin NKR-5-3AZ	*Enterococcus faecium*		5.2	59	
Microcin M	*Escherichia coli*	Impairs the cellular proton channel [17]	7.3	77	Chromosomally encoded, linear peptides that may carry a C-terminal posttranslational modification [18]
Microcin H47	*Escherichia coli*	Unknown	4.9	60	
**Subtype IIc: Circular**					
Lactococcin B	*Lactococcus lactis* *subsp. cremoris 9 B4*	Disrupt the proton motive force of the target cell by pore formation.	5.3	47	Cyclic peptides formed by the ligation of their N-terminus to the C-terminus via an amide bond (saposin fold) [16]
Enterocin B [19]	*Enterococcus faecium T136*		5.5	53	
**Subtype IId: Non-pediocin-like linear**					
Lacticin Q	*Lactococcus lactis QU 5*	Disrupt the proton motive force of the target cell by pore formation.	5.9	53	Other class II bacteriocins, including *sec*-dependent bacteriocins and leaderless bacteriocins [16]
Leucocin N	*Leuconostoc pseudomesenteroides QU 15*		3.7	32	
**Class III: Large proteins**					
***Heat-sensitive, hydrophilic peptides (>10 kDa)***					
**Subtype IIIa: Bacteriolytic**					
Helveticin V-1829	*Lactobacillus helveticus 1829*	bacteriolysins catalyze the hydrolysis of cell wall resulting in cell lysis			The C-terminal contain a recognition site for the target cell while the N-terminus has homology to endopeptidases involved in cell wall synthesis [20]
Lysostaphin	*Staphylococcus simulans* subsp. *staphylolyticus*		27	246	
**Subtype IIIb: Non-bacteriolytic**					
Helveticin J	*Lactobacillus helveticus 481*	Can disturb the glucose uptake by cells, starving them and also disturbs the membrane potential [19]	37	37	
Caseicin 80	*Lactobacillus casei*		42		
**Colicins, Pyocins, Salmocins**					
SalE1a	*Salmonella enterica*	Membrane pore formation	52.8		Colicin-like bacteriocins. Can be efficiently expressed in plants [21]
Colicin B	*Escherichia coli*		54.9	511	Subtype B—Use Ton system to penetrate the outer membrane of bacteria [22]
Colicin A	*Escherichia coli*		63.0	204	Subtype A—Use Tol system to penetrate the outer membrane of sensitive bacteria [22]
Colicin E2	*Escherichia coli*	DNase activity	59.6	581	
Pyocin S1	*Pseudomonas aeruginosa*		65.5	617	protease-sensitive “soluble” (S-type) Pyocins [23]
SalE2	*Salmonella enterica*		62.0		Colicin-like bacteriocins. Can be efficiently expressed in plants [21]
Pyocin R1-5	*Pseudomonas aeruginosa*	Depolarization of the cytoplasmic membrane			R-type pyocins resemble the contractile tails of Myoviridae bacteriophages, are rigid and non-flexuous particles [23]
**Class 1V: Circular proteins**					
**Heat-stable, hydrophobic lipid- or carbohydrate-conjugated complex proteins (** **∼** ***5.5–7.5 kDa)***					
Enterocin AS-48	*Enterococcus faecalis*	Insertion into cell membrane, resulting in membrane permeabilization [24]	7.14	70	cyclic peptides formed by the ligation of their N-terminus to the C-terminus via an amide bond [20]

**Table 2:** Pharmacological advantages and disadvantages of bacteriocins in comparison to antibiotics.

Characteristic	Bacteriocins	Antibiotic
Synthesis	Ribosomal (primary metabolite)	Enzymes (secondary metabolite)
Bioengineering	Highly amendable [16]	Not amendable
Spectrum of activity	Narrow (confined to closely related species)	Mainly broad
Potency	often in the nanomolar range [1]	Potent
Biocompatibility	Only toxic at high concentrations	Toxic
Working concentrations (MIC)	Lower (Often in the pico-nanomolar range)	Higher (usually in the micromolar range)
Chemical and thermal Stability	Tolerate a wide range of pH and temperature	Tolerate a narrow range PH and temperature
Adverse effects	None identified	Many
Diversity (i.e., in terms of size, microbial target, mode of action, etc.)	Broad	Narrow
Biodegradable	Completely metabolized in the human body	Persistent
Antibiofilm properties	Strong [38]	Resistance
Cost	High	Economically cost-effective
Purification	Complicated, low yield [25]	Possible, high yield
Specificity	Non-specific	Specific
Selectivity	Non-selective	Selective
Route of administration	protein degradation	Oral, IV, IM, topical, transdermal, nebulization etc.
Bioavailability	Size dependent	Good
Oral bioavailability	Poor	Good
Solubility	Low	Variable (low to high)
Metabolic stability	Low (Fast biotransformation)	Slow-fast biotransformation
Plasma stability	Low	Dependent on drug
Half Life	Low	Dependent on drug
Degradation	Enzymatic (proteolytic enzymes), [31]	Oxidative, Hydrolysis, photolytic, thermal
